# Can sociality facilitate learning of complex tasks? Lessons from bees and flowers

**DOI:** 10.1098/rstb.2021.0402

**Published:** 2023-03-13

**Authors:** Tamar Keasar, Odile Pourtallier, Eric Wajnberg

**Affiliations:** ^1^ Biology and Environment, University of Haifa, Oranim, Tivon 36006, Israel; ^2^ INRIA, Projet Hephaistos, 06902 Sophia Antipolis, France; ^3^ Inrae, 400 Route des Chappes, BP 167 06903 Sophia Antipolis Cedex, France

**Keywords:** evolutionary transition, eusociality, pollination, flower morphology, learning

## Abstract

The emergence of animal societies is a major evolutionary transition, but its implications for learning-dependent innovations are insufficiently understood. Bees, with lifestyles ranging from solitary to eusocial, are ideal models for exploring social evolution. Here, we ask how and why bees may acquire a new ‘technology’, foraging on morphologically complex flowers, and whether eusociality facilitates this technological shift. We consider ‘complex’ flowers that produce high food rewards but are difficult to access, *versus* ‘simple’ flowers offering easily accessible yet lower rewards. Complex flowers are less profitable than simple flowers to naive bees but become more rewarding after a learning period. We model how social bees optimally choose between simple and complex flowers over time, to maximize their colony's food balance. The model predicts no effect of colony size on the bees' flower choices. More foraging on complex flowers is predicted as colony longevity, its proportion of foragers, individual longevity and learning ability increase. Of these traits, only long-lived colonies and abundant foragers characterize eusocial bees. Thus, we predict that eusociality supports, but is not mandatory for, learning to exploit complex flowers. A re-analysis of a large published dataset of bee–flower interactions supports these conclusions. We discuss parallels between the evolution of insect sociality and other major transitions that provide scaffolds for learning innovations.

This article is part of the theme issue ‘Human socio-cultural evolution in light of evolutionary transitions’.

## 1. Introduction

The emergence of animal societies is a major evolutionary transition that is still insufficiently understood [1]. Bees are ideal models for testing ideas regarding the rise of animal sociality and its evolutionary implications. They form a speciose (approx. 20 000 species), monophyletic and widely distributed clade [2]. Sociality levels vary greatly among species [3], from solitary living to highly complex social groups. In solitary species, each female lays eggs and provisions her own offspring, typically during a single nesting season that lasts a few weeks. Subsocial and communal species form nesting aggregations, in which females share reproduction and nest defence to varying degrees. The highest level of sociality, termed eusociality, occurs in approximately 6% of bee species [4]. Colonies of eusocial bees survive for months or even years and comprise reproductive individuals (a queen and her sons) as well as sterile female workers (the queen's daughters). The colony functions as an inseparable superorganism. Namely, the queen mates and lays eggs, while the workers perform diverse tasks: forage for food (floral nectar and pollen) outside the colony, provision the brood with food, clean and guard inside the colony. These features, based on overlap of generations, reproductive division of labour and communal brood care, are considered the hallmarks of eusociality [5].

Many of the extant flowering plant species diversified during the same era (the past approx. 120 million years) as did bees [6,7], providing opportunities for coevolution [8]. Some of these plants, e.g. poppies and peonies, have radial, flat flowers with exposed anthers and nectaries. Such flowers are considered ‘simple’ because insects can land on them from any direction and can reach their food rewards easily. Simple flowers are visited by a wide range of pollinators, and hence are also known as ‘generalized’. Other plants, such as members of the pea and mint families, produce complex (also called ‘specialized’) flowers. Complex flowers are structurally defined as having floral parts of many different types, which often fuse to form elaborate structures [9]. Functionally, complex flowers possess morphological features that restrict the access of insect visitors to their nectar and pollen food rewards. These include: bilateral symmetry, which constrains the insects' landing angles on the corolla; fusion of petals to form long and/or narrow floral tubes; corollas that face sideways or downwards, thereby complicating landing; concealed nectaries; and poricidal anthers, which shed their pollen only after mechanical shaking [10–13]. At least two complexity-related traits, bilateral symmetry and the fusion of flower parts to form elaborate structures, increased in frequency over the course of angiosperm evolution [14,15].

Theory predicts higher nectar reward levels in complex flowers than in simple ones at any steady-state community of flowers and foragers [16,17]. The reasoning for this expectation is twofold: at the evolutionary level, since complex flowers can be pollinated by only a few specialized insects, they face a risk of pollen limitation. They should therefore be selected for higher nectar and pollen production rates to attract more visitors and reduce this risk [17,18]. At the proximate level, complex flowers are expected to be visited by fewer insects than simple flowers, and thus to have higher standing crops of food rewards because of reduced consumption by foragers. Such high food rewards may, in turn, increase the visitors’ tendency to transport pollen between flowers of the same species (flower constancy) and improve their pollination services [19]. Pollinators' visit constancy to complex, specialized flowers may even contribute to the conservation of rare species in plant communities [20].

Bees need to learn to efficiently handle the flowers that they feed on [21–23]. Handling flowers with complex morphologies is learned more slowly than handling simple ones [24]. Thus, new complex flower types may initially be less profitable to bees than familiar or simple flowers. Yet, new complex flowers become more profitable if bees persist and learn how to handle them, resulting in greatly reduced handling times and higher food intake rates. This is because a much larger improvement in handling proficiency occurs on complex than on simple flowers [13,24–26], and because of their often higher food rewards.

From the pollinators’ point of view, learning to feed on complex flowers and harvesting their food rewards is comparable to the uptake process of technological innovation. The process, although initially inefficient and time-consuming, eventually results in higher foraging success and should be favoured by natural selection. This is analogous to the challenge of understanding how prehistoric humans crossed initial barriers while adopting learning-dependent technological shifts, such as tool-making and farming. Learning ability is a heritable trait in insects, and strains with high learning abilities can be produced through artificial selection [27,28]. Bumblebees show a mild but persistent innate preference for complex flowers over simplified ones in flight-room experiments. Early training on complex artificial flowers, especially if paired with high reward, improves their learning of a second complex morphology that requires a different handling technique [29]. These findings suggest behavioural mechanisms that help overcome the initial learning costs of handling complex flowers. How these learning costs, and the mechanisms to reduce them, vary within and across pollinator species requires further study.

Here, we focus on bee pollinators and address two complementary questions: which life-history traits can promote feeding on complex flowers, and are those traits unique to eusocial bees? We model the acquisition of this new ‘technology’ for bees through learning and ask whether it is more likely to occur in eusocial species. Our model is guided by the following rationale: foragers on simple flowers attain a steady but low supply of food. Foragers on complex flowers, on the other hand, require much practice to learn how to access their concealed food rewards. On such complex flowers, inexperienced bees spend a long time handling each flower, initially achieving a low feeding rate (if they eventually get at the food reward) or even no feeding (if they fail to reach the food altogether). As bees gain experience, their handling time decreases and their probability of attaining the food reward increases. The model calculates how bees should optimally allocate their efforts between foraging on simple and complex flowers to maximize food intake for the entire colony. It predicts which life-history features shift the optimal allocation of foraging effort towards exploitation of complex flowers. We then evaluate whether these features are unique to eusocial bees.

## 2. The modelling framework

We built a discrete-time model, in which foragers from a eusocial bee colony (such as bumblebees) visit either simple or complex flowers. The colony contains *N* bees. A proportion *r* of them (for example, immatures and household workers) remain sedentary within the hive. The remaining *N*(1 − *r*) bees forage outside the hive for nectar and pollen.

The reward acquired on simple flowers is easy to obtain. For the sake of simplicity, the model assumes that simple flowers do not require any learning. On complex flowers, however, bees need to learn the correct handling technique to increase their reward harvesting rate. Once the learning period has been completed, the reward acquired on complex flowers is higher than on simple flowers. The model does not incorporate social learning; hence each forager needs to learn the complex flowers on its own.

As in previous studies [30–32], the learning curve on complex flowers is defined following the logistic equation:

$$c(t)=\frac{K}{1+e^{-(\alpha t-\beta )}}+m,$$

where *t* is the time spent on complex flowers, *m* is the minimal reward without learning, and *K* is the maximum progress achieved by means of the learning process; so *K* + *m* is the maximum reward after the learning process is done. The parameters *α* and *β* are respectively the shape and position parameters defining the learning process. Since an increase or decrease of *β* also corresponds to the shifting of the curve to the right or to the left, respectively, this parameter is considered as the bees' learning rate on complex flowers. Higher values of *β* correspond to lower learning rates. The inflection point on this logistic curve has an *x*-coordinate of *β/α.* Electronic supplementary material, S1 shows an example for the profitability of simple and complex flowers *versus* bee foraging experience, using the model's default parameter values (table 1):

**Table 1.** Variables used in the model and their default values.

parameter	meaning	default value
*T*	time steps to the horizon	100
*N*	total number of bees in the colony	100
*K*	parameter of the logistic learning curve	100
*m*	parameter of the logistic learning curve	10
*α*	parameter of the logistic learning curve	0.45
*β*	parameter of the logistic learning curve	10
*s*	reward on simple flowers	50
*q*	*per capita* consumption of the bees	30
steps	number of time steps	100
*r*	proportion of all bees that are sedentary in the hive	0.0
survival	proportion of bees that are still alive at the time horizon *T*	1.0

The model considers a time horizon, which corresponds to the time at which the beehive stops its activity. Each bee survives to the time horizon with a fixed probability, ‘survival’. Whenever a bee dies, it is replaced by a newly emerged, naive individual that has no information on how to forage on complex flowers.

All foragers initially visit simple flowers, and, at each time *t_i_*, *i* = 0, 1 · · · (*T* − 1), each bee can decide to visit simple or complex flowers during the next time step. The bees' decisions maximize the colony's overall net food intake across the time horizon. Any learning ability gained by a bee is never forgotten. Thus, if a bee decides to return to a simple flower, it retains its learning experience accumulated on complex flowers. Each bee in the colony is characterized by its experience acquired on complex flowers, which corresponds to the number of time steps it has foraged on such flowers.

The model calculates, for each time step, the fraction of foragers on simple and complex flowers that maximizes the colony's overall nectar intake. However, the maximization is constrained by the colony's need to maintain a positive nectar balance at each time step. We consider that each bee (whether sedentary or foraging) needs a quantity *q* of nectar to survive at each time step; thus the colony nectar stores must never fall below 0. A detailed description of the model, and the R code that implements it, are provided as electronic supplementary material, S2 and S3.

To predict how different life-history features may affect the bees' foraging on complex flowers, we manipulated the colony's time horizon (representing longevity), proportion of non-foraging bees (a proxy of communal foraging), and total hive size. We also tested the effects of the bees' learning ability of how to forage on complex flowers and of their individual survival probability.

## 3. The bee–flower interaction dataset

Roswell *et al*. [33] captured all (*n* = 18 698) bees from 109 plant species, along fixed transects. Five sampling rounds were conducted, in six plots in the eastern USA, during a single 11-week flowering season. Bees were sexed and determined to species (152 species), and their sociality level was recorded. We removed records of bee species with intermediate sociality levels, and retained only observations of species that are either solitary or eusocial. Thus, sociality was coded as a binary variable. We obtained the proboscis length (a common measure of body size; a continuous variable) for 66 of these bee species from Cariveau *et al*. [34]. Next, we compiled information on two proxies for the flowers’ handling difficulty: bilateral/radial symmetry (a binary variable; bilateral flowers are considered more complex (less accessible) than radial ones) and corolla tube length in millimetres (a continuous variable; long corollas are more complex than short ones). We obtained these data for 65 plant species in the database, based on Lovell & Lovell [35], Keller [36], Poindexter [37], Roberts [38], Barrow & Pickard [39], Harder [40], Desrochers *et al*. [41], Cresswell [42], Klinkhamer & de Jong [43], Comba *et al*. [44], Torres & Galetto [45], Caruso [46], Gawn [47], Junker *et al*. [48] and Junker & Parachnowitsch [49]. Additional information was retrieved from the websites https://linnet.geog.ubc.ca/biodiversity/eflora/, http://www.namethatplant.net/ and https://ucjeps.berkeley.edu/eflora. These pre-processing steps reduced our dataset to 10 856 observations that included information on bee sociality, bee body size and two measures of flower complexity. This reduced dataset includes 58 bee species and 67 plant species. Roswell's original published data are available at https://datadryad.org/stash/dataset/doi:10.5061/dryad.c3rr6q1. Our modified dataset is provided as electronic supplementary material, S4.

We tested for the effects of bee sociality, body size and their interaction on the symmetry and corolla depth of the visited flowers while accounting for the phylogenetic relatedness between the bees. All computations were done in R version 4.0.3 [50] using the packages ‘phyr’ [51] and ‘ape’ [52]. We used two phylogenetic generalized linear mixed (PGLMM) binomial models with a logit link function. In the first model, symmetry was treated as a binary response variable (either radial or bilateral). In the second model, corolla tubes were defined as either deep (longer than the median length of 3 mm) or shallow (≤3 mm); thus tube length was also treated as a binary variable. We took this approach because the continuous distribution of corolla tube lengths did not fit any of the theoretical distributions handled by the ‘lme4’ package [53]. Sociality and proboscis length were fixed factors in both models. The dataset contains non-independent observations because the same plots were sampled repeatedly, and because each bee species was recorded more than once in the field observations. To account for these repeated designs, we added sampling round and bee species as random factors to both models. We added the bees' genus-level phylogeny, based on Hedtke *et al*. [54], as an additional random factor to the PGLMM models, to incorporate the effect of phylogenetic relatedness on the bees’ flower choices.

## 4. Results

### (a) Colony longevity (time horizon)

**Figure 1.**
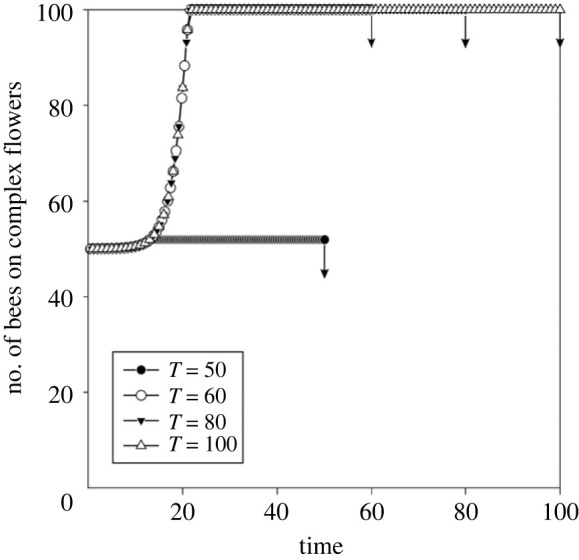
The optimal allocation of foragers to complex flowers for varying time horizons (colony longevity). The arrows indicate the end of the time horizon for the different values of *T*.

**Figure 2.**
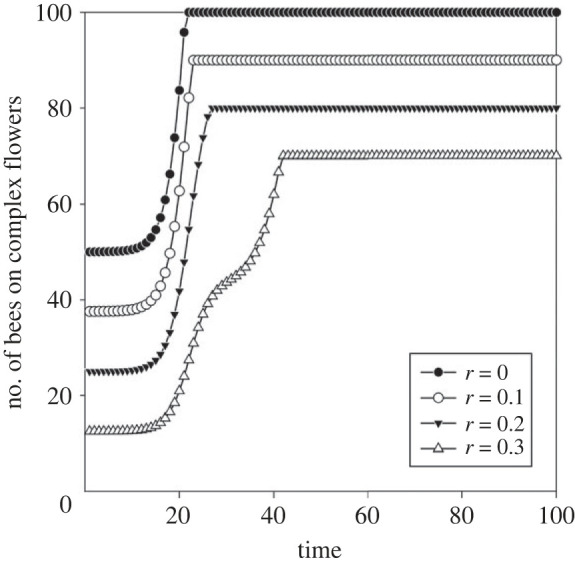
The optimal number of foragers on complex flowers for varying proportion of foragers in the colony (1 − *r*).

Using the model's default settings, one-half of the bees are predicted to shift from simple to complex flowers at the first time step (figure 1, *T* = 100). Progressively, increasing numbers of bees visit complex flowers. Since the default number of bees in the colony is set to 100, the *y*-axis in figure 1 (as well as in figures 2, 4, 5) can also be interpreted as the percentage of individuals that visit the complex flowers. The optimal switch to complex flowers starts as soon as there are enough resources in the hive to afford the learning period of the bees that start foraging on complex flowers. After the learning period, these bees bring back more food to the hive than those visiting simple flowers. This results in all bees eventually foraging on complex flowers only.

The optimal strategy changes if the lifespan of the colony is reduced. When the reduction is mild (figure 1, *T* = 60 or *T* = 80), the shift to foraging on complex flowers is unaffected, but the time remaining for the colony to exploit the complex flowers decreases. At even shorter lifespan values (*T* = 50), there is insufficient time left to exploit the complex flowers after their handling technique had been learnt. In this case, the best strategy is to forage on simple and complex flowers about equally throughout the colony's life.

### (b) The fraction of non-foraging bees

As the proportion of non-foragers in the colony increases, the optimal total fraction of foragers that visit complex flowers decreases, and the switch from simple to complex flowers is delayed (figure 2). This is because most of the foraging effort is needed to maintain a positive energy budget for the colony by visiting simple flowers. This leads to a lower investment in learning to handle the complex flowers. Eventually, however, all the bees that forage outside of the colony switch to feeding on complex flowers.

### (c) Colony size

**Figure 3.**
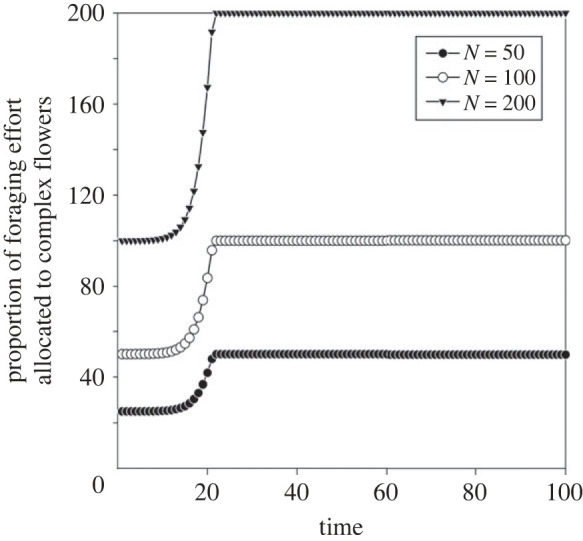
The optimal allocation of foraging effort on complex flowers for varying colony sizes (*N*).

Everything else being equal, the total number of bees in the colony does not affect the timing of the transition to foraging on complex flowers (figure 3). This reflects that, in the model, we explicitly took into account a balance between two opposing processes: larger colonies have more workers that are available to visit complex flowers, but also need more food to maintain a positive energy budget (favouring visits to simple flowers). This is also due to the fact that the model is linear, which means that potential interactions between bees are explicitly not considered. With the default parameter values, the model predicts all bees in the colony to shift to complex flowers at time step 22, regardless of colony size. For small colonies, the model predicts the bees' optimal proportions of foraging time on simple and complex flowers, rather than the optimal number of foragers on each flower type. This would be the case, for example, if there is only one bee in the colony (equivalent to a solitary bee).

### (d) Learning rates

**Figure 4.**
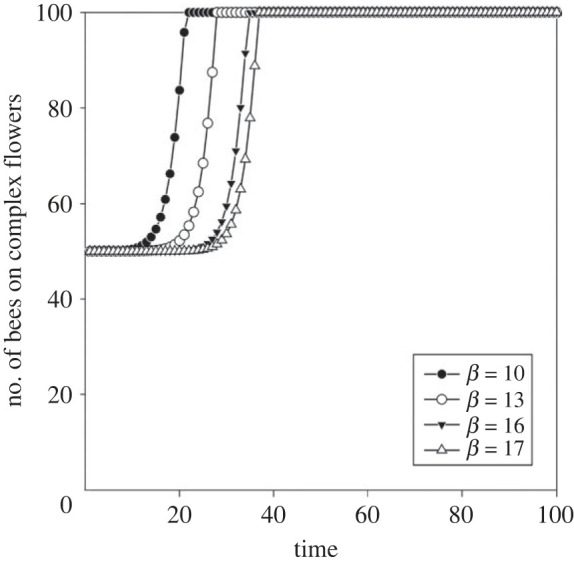
The optimal number of foragers on complex flowers at varying learning rates *β*.

Increasing the value of *β (*from 10 to 13, 16 and 17) while keeping the default value for *α* shifted the inflection point of the learning curve to the right, i.e. reduced the bees’ learning rates. Better learners shifted earlier from simple to complex flowers than slower learners (figure 4), because they are quicker to learn to feed efficiently on complex flowers.

### (e) Individual longevity

**Figure 5.**
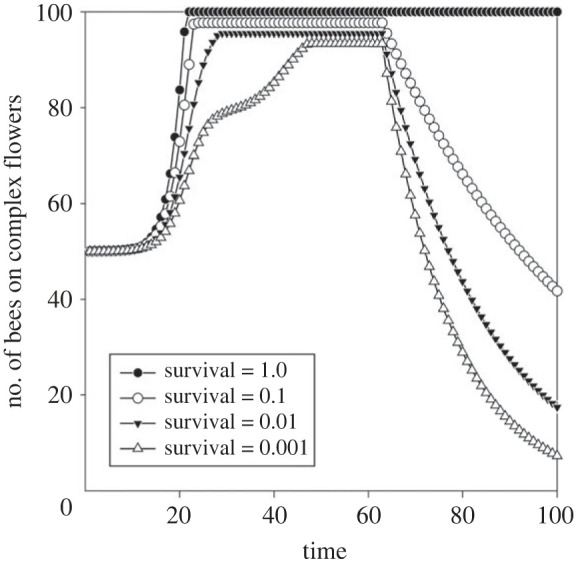
The optimal number of foragers on complex flowers at varying probabilities of forager survival (=individual lifespan).

When the foragers’ survival probability declines, after a while the newly emerged individuals do not have sufficient time to learn how to forage on complex flowers, and thus gain higher food rewards by remaining on simple flowers. This explains the decrease in the cumulated numbers of bees choosing complex flowers on the right side of figure 5.

### (f) The bee–flower interaction dataset

We considered bilateral symmetry and long corolla tubes as proxies for complex flowers, whereas radially symmetrical flowers with short corolla tubes were considered to be simple. Of the 10 856 bee–flower interactions from Roswell *et al*. [33] that we analysed, 5786 involved flowers with radial symmetry and 5070 involved flowers with bilateral symmetry. None of the flowers was asymmetrical. Eusocial and solitary bees made 0.517 ± 0.067 (mean ± s.e., *n* = 20 species) and 0.315 ± 0.059 (38 species) of their visits to bilateral flowers, respectively. Eusocial and solitary species were similar in size (mean proboscis lengths: 4.091 ± 0.832 mm for the social species and 3.893 ± 0.312 mm for the solitary ones). The smallest bees in the dataset (proboscis length between 0 and 3 mm) made most (68.8%) of the visits to the radial flowers, while the largest bees (proboscis length 9–12 mm) performed most (57.9%) of the visits to bilateral flowers. The PGLMM model revealed a significant effect of body size (Wald's statistic = 6.361*, p* < 0.001), alone and in interaction with sociality (Wald's = −4.246*, p* < 0.001), on the symmetry of visited flowers, while sociality was non-significant as a main factor (Wald's = 0.184, *p =* 0.854).

Eusocial bees visited flowers with a mean ± s.e. corolla depth of 7.472 ± 1.108 mm, while the mean corolla depth visited by the solitary species was 4.746 ± 0.583 mm. The smallest bees (proboscis less than 3 mm) made the lowest proportion of their visits to long-tubed flowers (0.337 ± 0.058). The largest bees (proboscis greater than 9 mm) visited long-tubed flowers most often (0.709 ± 0.108 of their visits). Body size significantly affected the tube length of the visited flowers (PGLMM, Wald's = 5.676, *p* < 0.001). Sociality tended to increase the bees' tendency to visit long-tubed flowers, but this trend did not reach statistical significance (Wald's = 1.7267, *p* = 0.084), nor did its interaction with body size have a significant effect (Wald's = −0.208, *p* = 0.835).

## 5. Discussion

Using a theoretical model of bee foraging, we predicted the effects of several life-history parameters on bees' tendency to visit morphologically complex flowers. The variables manipulated in the model, and their predicted effects on the bees’ foraging choices, are summarized in table 2.

**Table 2.** Model variables manipulated in the model, and the expected effects on visits to complex flowers.

tested variable	biological interpretation	model prediction: increasing this parameter should lead to:	rationale
*T*	colony longevity	more visits to complex flowers	after learning to handle complex flowers, more time is available for the colony to reap the benefits
*r*	proportion of bees that do not leave the hive	fewer visits to complex flowers	reduced communal foraging; more visits to simple flowers needed to retain positive energy balance
*N*	colony size	no effect on visits to complex flowers	increasing the number of bees increases the overall harvested food, but also the amount of food needed to maintain a positive nectar balance
*β*	learning rate	fewer visits to complex flowers	learning period on complex flowers is increased, reducing their foraging benefit
survival	individual longevity	more visits to complex flowers	after learning to handle complex flowers, more time is available for each forager to reap the benefits

Key aspects of eusocial bee colonies are overlapping generations, reproductive caste differentiation and communal care of the brood by the non-reproductive adult workers. Our model predicts that some of these features can help bees overcome the initial costs of learning to access complex flowers, and increase the colony's overall food intake. The existence of overlapping generations in the colony favours long-lived colonies. The reserves built up by previous generations of foragers in such colonies allow the bees to spend their time learning the handling of complex flowers. This, in turn, increases the colony's benefit from learning how to forage on complex flowers. Reproductive division of labour and communal brood care require a high colony-level foraging effort, to provision the brood produced by the queen. Dedicating a high proportion of the workers' time to foraging, according to the model, can increase the optimal effort allocated to complex flowers: sufficient foragers are available on simple flowers to prevent colony starvation, while others gain experience on the complex flowers. The model also identifies high longevity and learning abilities of individual adult foragers as important life-history variables that favour learning how to exploit complex flowers efficiently. As far as we are aware, there is no evidence for greater longevity in adult workers of eusocial bees compared with solitary females. Body (and brain) sizes predict better associative learning abilities in comparisons across bee species, whereas sociality does not [55]. Hence, both solitary and social bees should optimally feed on complex flowers, provided they are sufficiently good learners, and long-lived enough to learn how to forage on such flowers.

Taken together, our modelling results suggest some life-history traits that favour learning how to forage on complex flowers and are linked to sociality, while other salient life-history traits seem to be independent of social organization. This implies that eusociality can facilitate shifts from simple to complex flowers, but is not mandatory for such shifts to occur. In support of this idea, social and solitary bees differ in other aspects of foraging behaviour, such as higher flower specialization in solitary species ([56,57]; but see [58]). We analysed a large dataset of bee–flower interactions to test to what degree bee sociality predicts foraging on complex flowers, using two indicators of flower complexity: bilateral (complex) *versus* radial (simple) symmetry and deep (complex) *versus* shallow (simple) corolla tubes. Indeed, we find that both solitary and eusocial bees visit bilateral and deep-tubed (complex) flowers. We also find that sociality interacts with body size to enhance foraging on bilaterally symmetrical flowers and that sociality tends to increase foraging on long-tubed flowers. As in many earlier studies, our empirical dataset shows that larger (longer-tongued) bees visit flowers with deeper corolla tubes. This has previously been attributed to size-matching between proboscis and corolla lengths [59], which improves foraging efficiency [60]. Our model suggests an additional interpretation to the empirically observed correlation between bee size and flower depth: learning ability improves with body size across bee species [55], and therefore larger bees can gain higher long-term benefits from investing foraging efforts in complex flowers.

The emergence of novel levels of individuality is a recurrent theme in the history of life [61]. Biological units that previously existed as independent individuals (e.g. individual bees) are incorporated within a higher level of organization (such as a bee colony, which becomes a unit of selection). New levels of individuality form during such evolutionary transitions. How such major transitions occur is a topic of sustained discussion in evolutionary theory [62,63]. Our model suggests a novel hypothesis for a specific evolutionary transition, the emergence of eusociality in insects, which complements and expands the currently available explanations. The model links a change in ecological conditions, the radiation of morphological complex flowers, with the social evolution of their pollinators. In other words, we propose that life-history traits correlated with social living allow a subset of foragers to cross local minima on the fitness landscape *en route* to the fitness peaks, as they gradually learn to exploit highly profitable complex flowers.

Other contributions to this special issue discuss other ecological changes as scaffolds that help overcome local minima during evolutionary transitions, with an emphasis on human cultural evolution. Szilágyi *et al*. [64] suggest that the drying climate in East Africa two million years ago triggered a shift in the diet of early humans, towards group-scavenging on large animal carcasses. This shift, in turn, favoured the development of language and tool use. Similarly, a period of benign climatic conditions in the Levant during the Natufian era may have facilitated the transition of human societies from nomadic to sedentary, paving the way for the development of agriculture [65]. Rainey [66] generalizes the idea of ‘ecological scaffolding’ to additional evolutionary transitions that follow novel ecological conditions, including a potential future transition that may combine humans and computers into a new unit of selection. By extending these ideas to non-human evolution, we hope to stimulate further research into the rare events that overcome barriers to evolutionary transitions.

## Data Availability

The paper describes a mathematical model and a statistical re-analysis of published data. The model's description and code are provided in the electronic supplementary material [67]. The data used for analyses are also provided in the electronic supplementary material.
